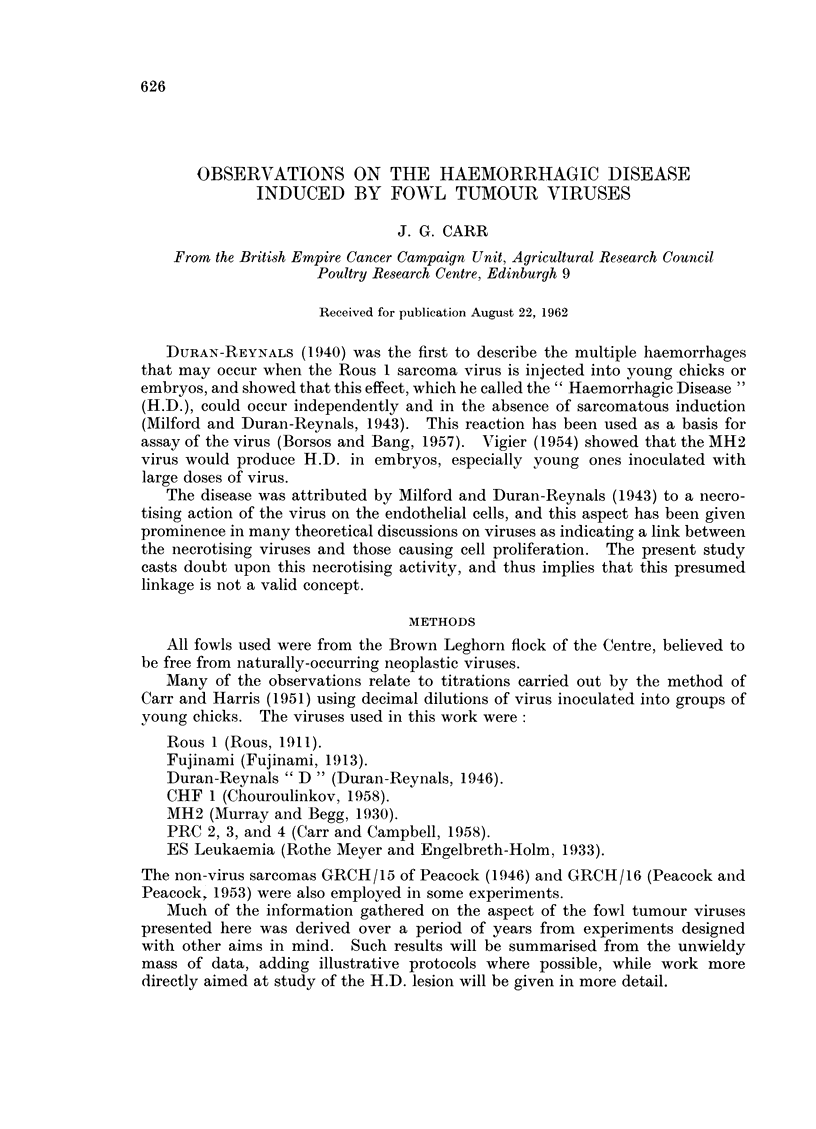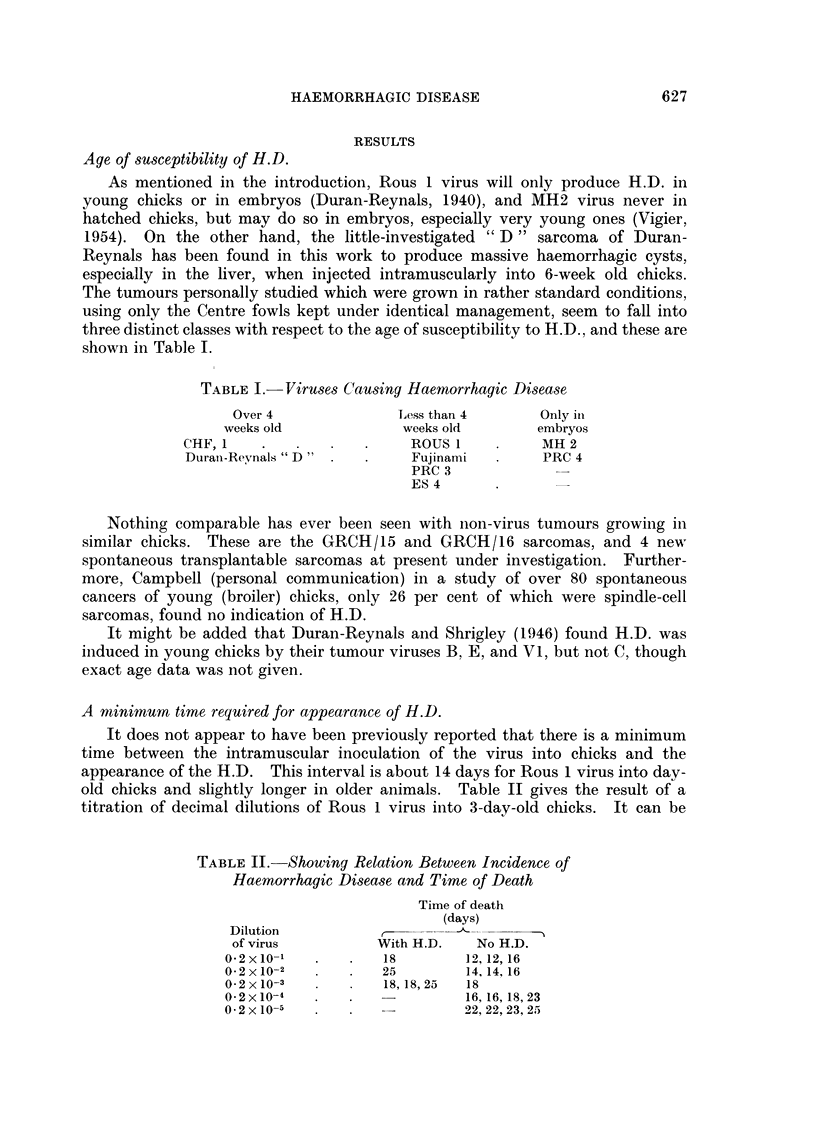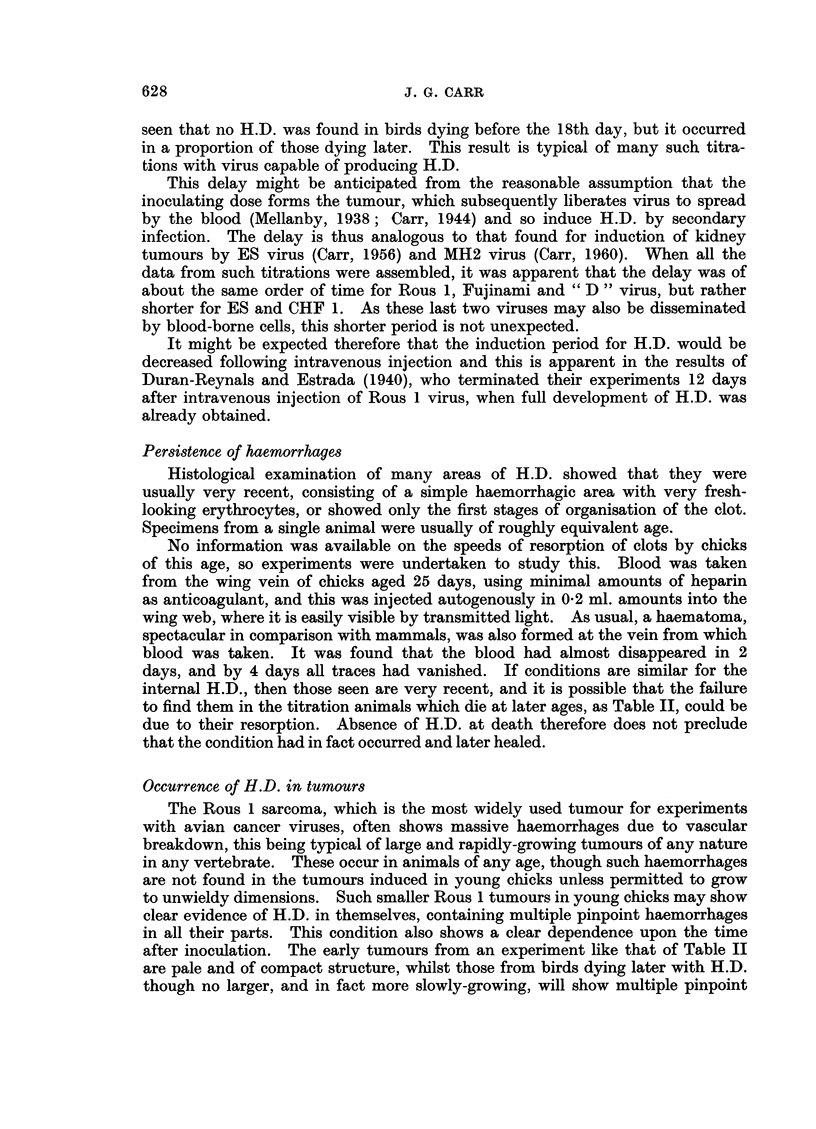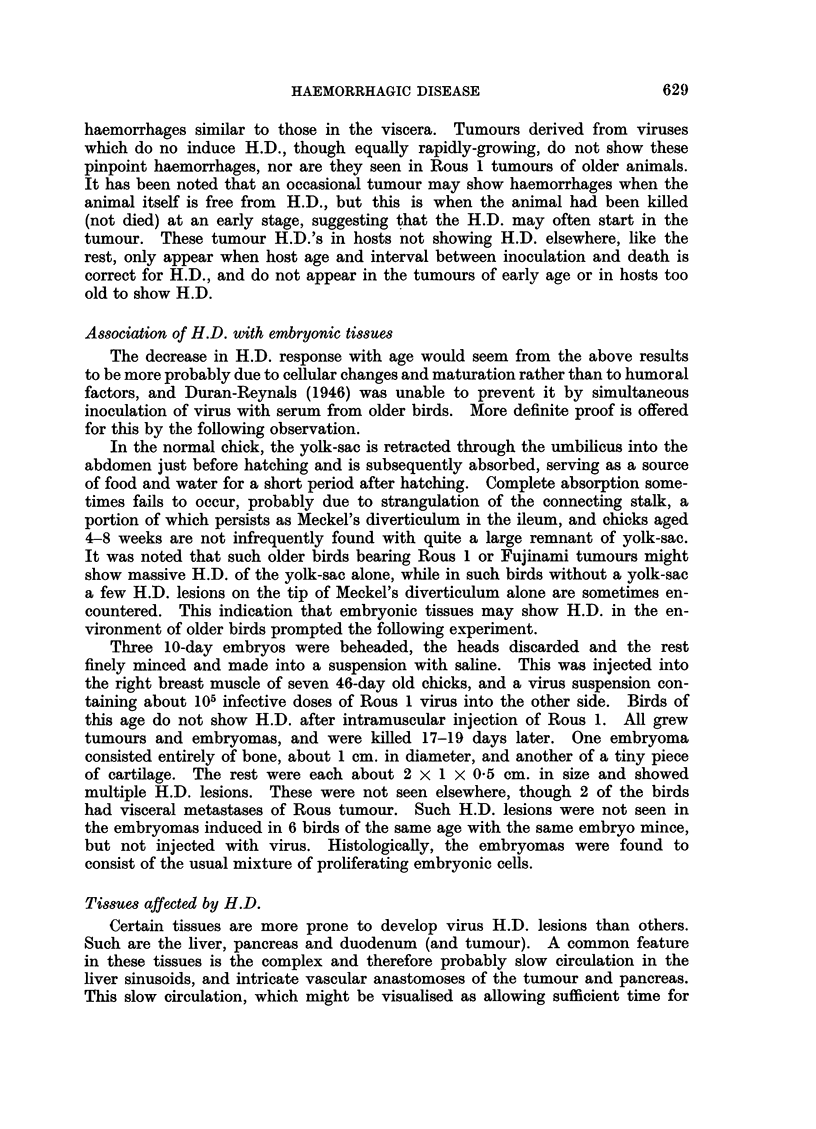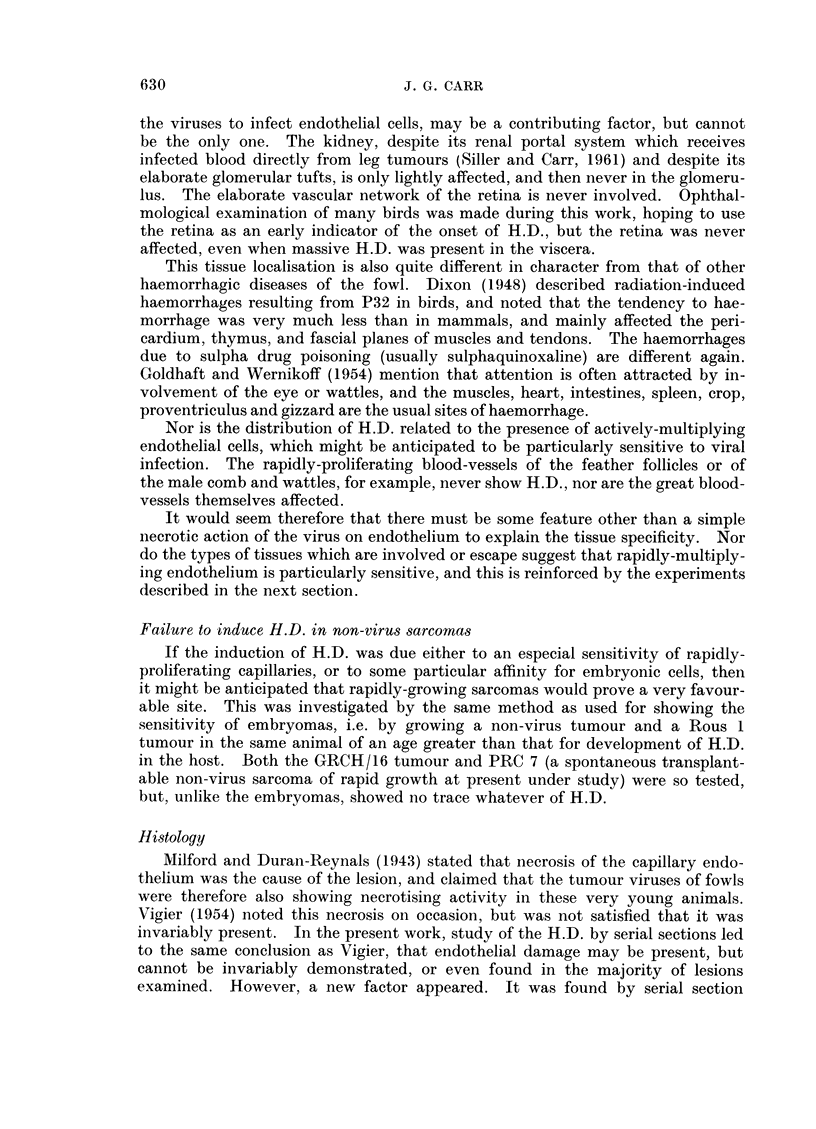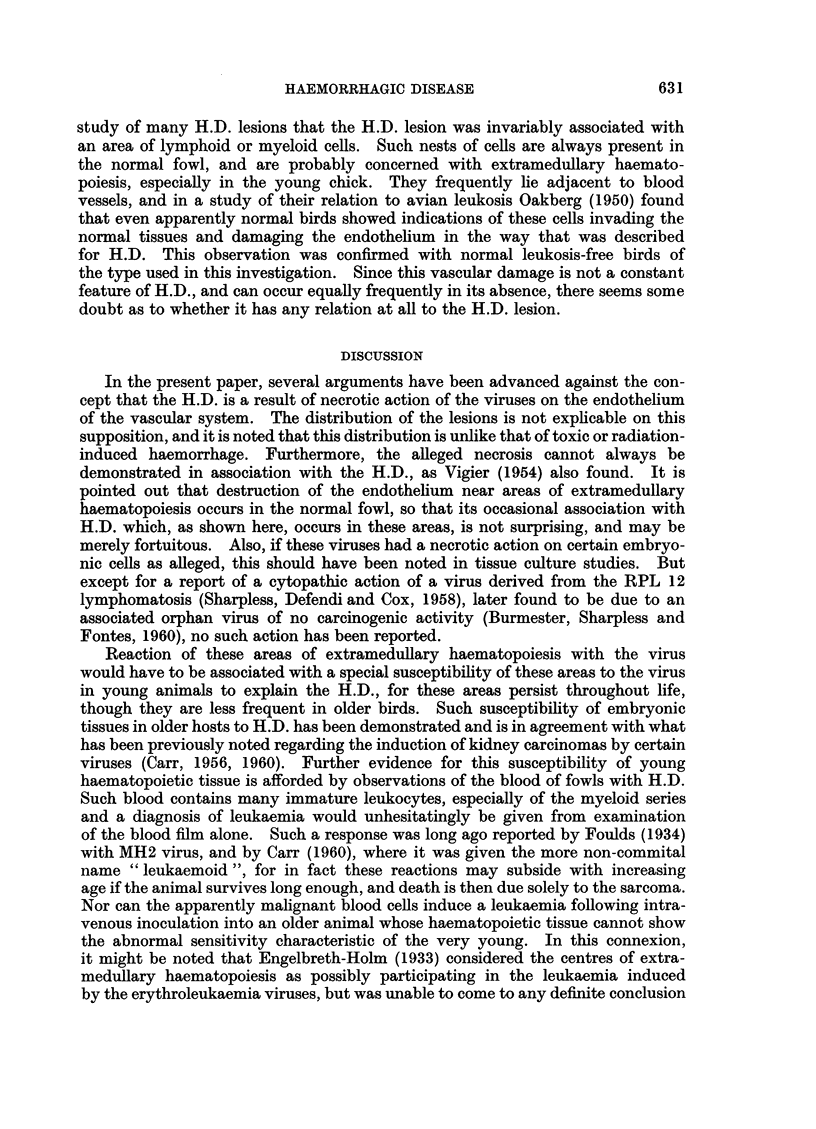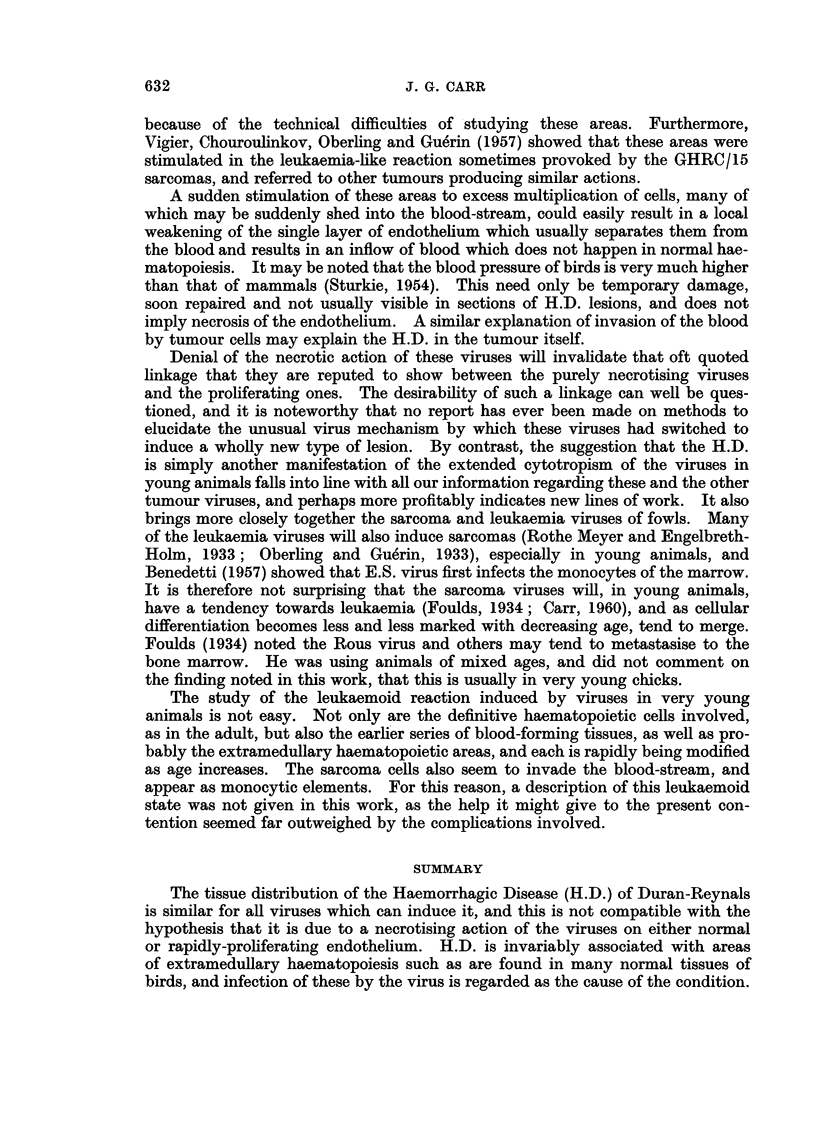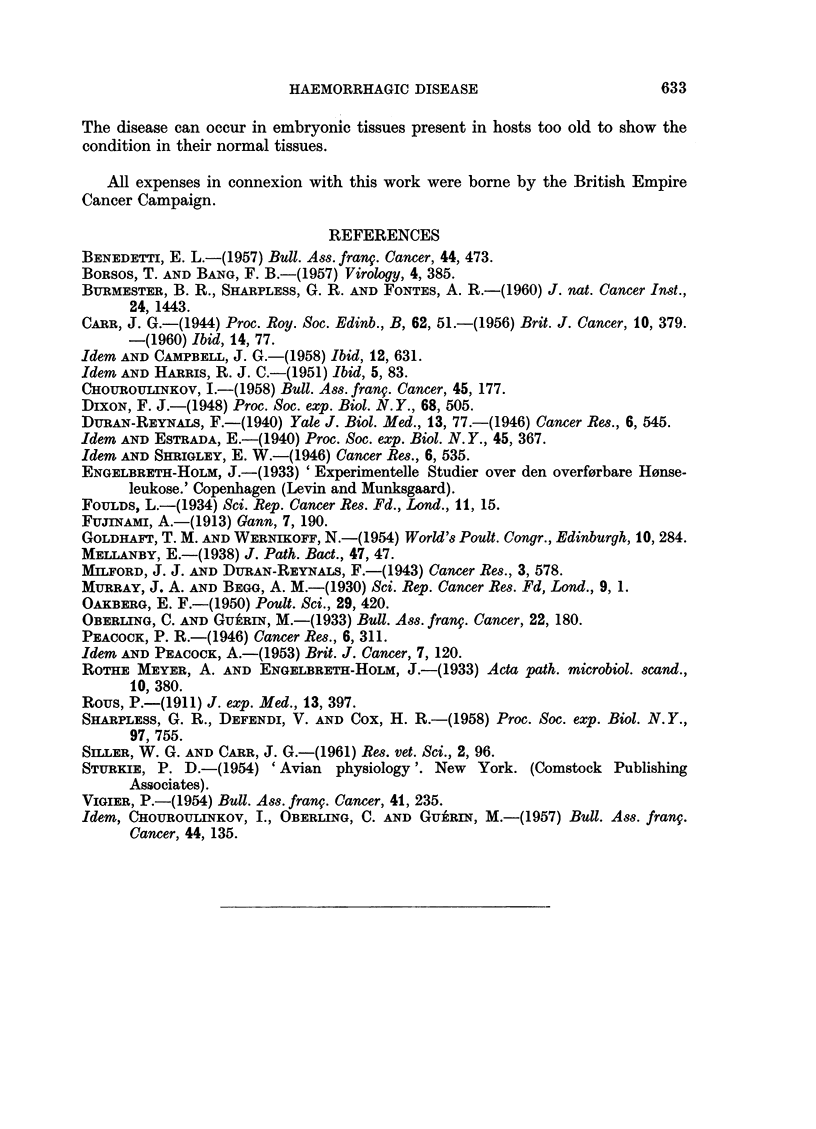# Observations on the Haemorrhagic Disease Induced by Fowl Tumour Viruses

**DOI:** 10.1038/bjc.1962.74

**Published:** 1962-12

**Authors:** J. G. Carr


### OBSERVATIONS ON THE HAEMORRHAGIC DISEASE INDUCED BY FOWL TUMOUR VIRUSES

J. G. CARR

*From the British Empire Cancer Campaign Unit, Agricultural Research Council Poultry Research Centre, Edinburgh* 9

Received for publication August 22, 1962

DURAN-REYNALS (1940) was the first to describe the multiple haemorrhages that may occur when the Rous 1 sarcoma virus is injected into young chicks or embryos, and showed that this effect, which he called the " Haemorrhagic Disease " (H.D.), could occur independently and in the absence of sarcomatous induction (Milford and Duran-Reynals, 1943). This reaction has been used as a basis for assay of the virus (Borsos and Bang, 1957). Vigier (1954) showed that the MH2 virus would produce H.D. in embryos, especially young ones inoculated with large doses of virus.

The disease was attributed by Milford and Duran-Reynals (1943) to a necrotising action of the virus on the endothelial cells, and this aspect has been given prominence in many theoretical discussions on viruses as indicating a link between the necrotising viruses and those causing cell proliferation. The present study casts doubt upon this necrotising activity, and thus implies that this presumed linkage is not a valid concept.

### METHODS

All fowls used were from the Brown Leghorn flock of the Centre, believed to be free from naturally-occurring neoplastic viruses.

Many of the observations relate to titrations carried out by the method of Carr and Harris (1951) using decimal dilutions of virus inoculated into groups of young chicks. The viruses used in this work were :

Rous 1 (Rous, 1911).
Fujinami (Fujinami, 1913).
Duran-Reynals " D " (Duran-Reynals, 1946).
CHF 1 (Chouroulinkov, 1958).
MH2 (Murray and Begg, 1930).
PRC 2, 3, and 4 (Carr and Campbell, 1958).
ES Leukaemia (Rothe Meyer and Engelbreth-Holm, 1933).

The non-virus sarcomas GRCH/15 of Peacock (1946) and GRCH/16 (Peacock and Peacock, 1953) were also employed in some experiments.

Much of the information gathered on the aspect of the fowl tumour viruses presented here was derived over a period of years from experiments designed with other aims in mind. Such results will be summarised from the unwieldy mass of data, adding illustrative protocols where possible, while work more directly aimed at study of the H.D. lesion will be given in more detail.

### RESULTS

*Age of susceptibility of H.D.*

As mentioned in the introduction, Rous 1 virus will only produce H.D. in young chicks or in embryos (Duran-Reynals, 1940), and MH2 virus never in hatched chicks, but may do so in embryos, especially very young ones (Vigier, 1954). On the other hand, the little-investigated " D " sarcoma of Duran-Reynals has been found in this work to produce massive haemorrhagic cysts, especially in the liver, when injected intramuscularly into 6-week old chicks. The tumours personally studied which were grown in rather standard conditions, using only the Centre fowls kept under identical management, seem to fall into three distinct classes with respect to the age of susceptibility to H.D., and these are shown in Table I.

TABLE I.—*Viruses Causing Haemorrhagic Disease*

| Over 4 weeks old | Less than 4 weeks old | Only in embryos |
|---|---|---|
| CHF, 1 | ROUS 1 | MH 2 |
| Duran-Reynals " D " | Fujinami | PRC 4 |
| | PRC 3 | — |
| | ES 4 | — |

Nothing comparable has ever been seen with non-virus tumours growing in similar chicks. These are the GRCH/15 and GRCH/16 sarcomas, and 4 new spontaneous transplantable sarcomas at present under investigation. Furthermore, Campbell (personal communication) in a study of over 80 spontaneous cancers of young (broiler) chicks, only 26 per cent of which were spindle-cell sarcomas, found no indication of H.D.

It might be added that Duran-Reynals and Shrigley (1946) found H.D. was induced in young chicks by their tumour viruses B, E, and V1, but not C, though exact age data was not given.

*A minimum time required for appearance of H.D.*

It does not appear to have been previously reported that there is a minimum time between the intramuscular inoculation of the virus into chicks and the appearance of the H.D. This interval is about 14 days for Rous 1 virus into day-old chicks and slightly longer in older animals. Table II gives the result of a titration of decimal dilutions of Rous 1 virus into 3-day-old chicks. It can be

TABLE II.—*Showing Relation Between Incidence of Haemorrhagic Disease and Time of Death*

| Dilution of virus | Time of death (days) | |
|---|---|---|
| | With H.D. | No H.D. |
| $0{\cdot}2 \times 10^{-1}$ | 18 | 12, 12, 16 |
| $0{\cdot}2 \times 10^{-2}$ | 25 | 14, 14, 16 |
| $0{\cdot}2 \times 10^{-3}$ | 18, 18, 25 | 18 |
| $0{\cdot}2 \times 10^{-4}$ | — | 16, 16, 18, 23 |
| $0{\cdot}2 \times 10^{-5}$ | — | 22, 22, 23, 25 |

seen that no H.D. was found in birds dying before the 18th day, but it occurred in a proportion of those dying later. This result is typical of many such titrations with virus capable of producing H.D.

This delay might be anticipated from the reasonable assumption that the inoculating dose forms the tumour, which subsequently liberates virus to spread by the blood (Mellanby, 1938; Carr, 1944) and so induce H.D. by secondary infection. The delay is thus analogous to that found for induction of kidney tumours by ES virus (Carr, 1956) and MH2 virus (Carr, 1960). When all the data from such titrations were assembled, it was apparent that the delay was of about the same order of time for Rous 1, Fujinami and " D " virus, but rather shorter for ES and CHF 1. As these last two viruses may also be disseminated by blood-borne cells, this shorter period is not unexpected.

It might be expected therefore that the induction period for H.D. would be decreased following intravenous injection and this is apparent in the results of Duran-Reynals and Estrada (1940), who terminated their experiments 12 days after intravenous injection of Rous 1 virus, when full development of H.D. was already obtained.

*Persistence of haemorrhages*

Histological examination of many areas of H.D. showed that they were usually very recent, consisting of a simple haemorrhagic area with very fresh-looking erythrocytes, or showed only the first stages of organisation of the clot. Specimens from a single animal were usually of roughly equivalent age.

No information was available on the speeds of resorption of clots by chicks of this age, so experiments were undertaken to study this. Blood was taken from the wing vein of chicks aged 25 days, using minimal amounts of heparin as anticoagulant, and this was injected autogenously in 0·2 ml. amounts into the wing web, where it is easily visible by transmitted light. As usual, a haematoma, spectacular in comparison with mammals, was also formed at the vein from which blood was taken. It was found that the blood had almost disappeared in 2 days, and by 4 days all traces had vanished. If conditions are similar for the internal H.D., then those seen are very recent, and it is possible that the failure to find them in the titration animals which die at later ages, as Table II, could be due to their resorption. Absence of H.D. at death therefore does not preclude that the condition had in fact occurred and later healed.

*Occurrence of H.D. in tumours*

The Rous 1 sarcoma, which is the most widely used tumour for experiments with avian cancer viruses, often shows massive haemorrhages due to vascular breakdown, this being typical of large and rapidly-growing tumours of any nature in any vertebrate. These occur in animals of any age, though such haemorrhages are not found in the tumours induced in young chicks unless permitted to grow to unwieldy dimensions. Such smaller Rous 1 tumours in young chicks may show clear evidence of H.D. in themselves, containing multiple pinpoint haemorrhages in all their parts. This condition also shows a clear dependence upon the time after inoculation. The early tumours from an experiment like that of Table II are pale and of compact structure, whilst those from birds dying later with H.D. though no larger, and in fact more slowly-growing, will show multiple pinpoint haemorrhages similar to those in the viscera. Tumours derived from viruses which do no induce H.D., though equally rapidly-growing, do not show these pinpoint haemorrhages, nor are they seen in Rous 1 tumours of older animals. It has been noted that an occasional tumour may show haemorrhages when the animal itself is free from H.D., but this is when the animal had been killed (not died) at an early stage, suggesting that the H.D. may often start in the tumour. These tumour H.D.'s in hosts not showing H.D. elsewhere, like the rest, only appear when host age and interval between inoculation and death is correct for H.D., and do not appear in the tumours of early age or in hosts too old to show H.D.

*Association of H.D. with embryonic tissues*

The decrease in H.D. response with age would seem from the above results to be more probably due to cellular changes and maturation rather than to humoral factors, and Duran-Reynals (1946) was unable to prevent it by simultaneous inoculation of virus with serum from older birds. More definite proof is offered for this by the following observation.

In the normal chick, the yolk-sac is retracted through the umbilicus into the abdomen just before hatching and is subsequently absorbed, serving as a source of food and water for a short period after hatching. Complete absorption sometimes fails to occur, probably due to strangulation of the connecting stalk, a portion of which persists as Meckel's diverticulum in the ileum, and chicks aged 4–8 weeks are not infrequently found with quite a large remnant of yolk-sac. It was noted that such older birds bearing Rous 1 or Fujinami tumours might show massive H.D. of the yolk-sac alone, while in such birds without a yolk-sac a few H.D. lesions on the tip of Meckel's diverticulum alone are sometimes encountered. This indication that embryonic tissues may show H.D. in the environment of older birds prompted the following experiment.

Three 10-day embryos were beheaded, the heads discarded and the rest finely minced and made into a suspension with saline. This was injected into the right breast muscle of seven 46-day old chicks, and a virus suspension containing about $10^5$ infective doses of Rous 1 virus into the other side. Birds of this age do not show H.D. after intramuscular injection of Rous 1. All grew tumours and embryomas, and were killed 17–19 days later. One embryoma consisted entirely of bone, about 1 cm. in diameter, and another of a tiny piece of cartilage. The rest were each about $2 \times 1 \times 0{\cdot}5$ cm. in size and showed multiple H.D. lesions. These were not seen elsewhere, though 2 of the birds had visceral metastases of Rous tumour. Such H.D. lesions were not seen in the embryomas induced in 6 birds of the same age with the same embryo mince, but not injected with virus. Histologically, the embryomas were found to consist of the usual mixture of proliferating embryonic cells.

*Tissues affected by H.D.*

Certain tissues are more prone to develop virus H.D. lesions than others. Such are the liver, pancreas and duodenum (and tumour). A common feature in these tissues is the complex and therefore probably slow circulation in the liver sinusoids, and intricate vascular anastomoses of the tumour and pancreas. This slow circulation, which might be visualised as allowing sufficient time for the viruses to infect endothelial cells, may be a contributing factor, but cannot be the only one. The kidney, despite its renal portal system which receives infected blood directly from leg tumours (Siller and Carr, 1961) and despite its elaborate glomerular tufts, is only lightly affected, and then never in the glomerulus. The elaborate vascular network of the retina is never involved. Ophthalmological examination of many birds was made during this work, hoping to use the retina as an early indicator of the onset of H.D., but the retina was never affected, even when massive H.D. was present in the viscera.

This tissue localisation is also quite different in character from that of other haemorrhagic diseases of the fowl. Dixon (1948) described radiation-induced haemorrhages resulting from P32 in birds, and noted that the tendency to haemorrhage was very much less than in mammals, and mainly affected the pericardium, thymus, and fascial planes of muscles and tendons. The haemorrhages due to sulpha drug poisoning (usually sulphaquinoxaline) are different again. Goldhaft and Wernikoff (1954) mention that attention is often attracted by involvement of the eye or wattles, and the muscles, heart, intestines, spleen, crop, proventriculus and gizzard are the usual sites of haemorrhage.

Nor is the distribution of H.D. related to the presence of actively-multiplying endothelial cells, which might be anticipated to be particularly sensitive to viral infection. The rapidly-proliferating blood-vessels of the feather follicles or of the male comb and wattles, for example, never show H.D., nor are the great blood-vessels themselves affected.

It would seem therefore that there must be some feature other than a simple necrotic action of the virus on endothelium to explain the tissue specificity. Nor do the types of tissues which are involved or escape suggest that rapidly-multiplying endothelium is particularly sensitive, and this is reinforced by the experiments described in the next section.

*Failure to induce H.D. in non-virus sarcomas*

If the induction of H.D. was due either to an especial sensitivity of rapidly-proliferating capillaries, or to some particular affinity for embryonic cells, then it might be anticipated that rapidly-growing sarcomas would prove a very favourable site. This was investigated by the same method as used for showing the sensitivity of embryomas, i.e. by growing a non-virus tumour and a Rous 1 tumour in the same animal of an age greater than that for development of H.D. in the host. Both the GRCH/16 tumour and PRC 7 (a spontaneous transplantable non-virus sarcoma of rapid growth at present under study) were so tested, but, unlike the embryomas, showed no trace whatever of H.D.

*Histology*

Milford and Duran-Reynals (1943) stated that necrosis of the capillary endothelium was the cause of the lesion, and claimed that the tumour viruses of fowls were therefore also showing necrotising activity in these very young animals. Vigier (1954) noted this necrosis on occasion, but was not satisfied that it was invariably present. In the present work, study of the H.D. by serial sections led to the same conclusion as Vigier, that endothelial damage may be present, but cannot be invariably demonstrated, or even found in the majority of lesions examined. However, a new factor appeared. It was found by serial section study of many H.D. lesions that the H.D. lesion was invariably associated with an area of lymphoid or myeloid cells. Such nests of cells are always present in the normal fowl, and are probably concerned with extramedullary haematopoiesis, especially in the young chick. They frequently lie adjacent to blood vessels, and in a study of their relation to avian leukosis Oakberg (1950) found that even apparently normal birds showed indications of these cells invading the normal tissues and damaging the endothelium in the way that was described for H.D. This observation was confirmed with normal leukosis-free birds of the type used in this investigation. Since this vascular damage is not a constant feature of H.D., and can occur equally frequently in its absence, there seems some doubt as to whether it has any relation at all to the H.D. lesion.

### DISCUSSION

In the present paper, several arguments have been advanced against the concept that the H.D. is a result of necrotic action of the viruses on the endothelium of the vascular system. The distribution of the lesions is not explicable on this supposition, and it is noted that this distribution is unlike that of toxic or radiation-induced haemorrhage. Furthermore, the alleged necrosis cannot always be demonstrated in association with the H.D., as Vigier (1954) also found. It is pointed out that destruction of the endothelium near areas of extramedullary haematopoiesis occurs in the normal fowl, so that its occasional association with H.D. which, as shown here, occurs in these areas, is not surprising, and may be merely fortuitous. Also, if these viruses had a necrotic action on certain embryonic cells as alleged, this should have been noted in tissue culture studies. But except for a report of a cytopathic action of a virus derived from the RPL 12 lymphomatosis (Sharpless, Defendi and Cox, 1958), later found to be due to an associated orphan virus of no carcinogenic activity (Burmester, Sharpless and Fontes, 1960), no such action has been reported.

Reaction of these areas of extramedullary haematopoiesis with the virus would have to be associated with a special susceptibility of these areas to the virus in young animals to explain the H.D., for these areas persist throughout life, though they are less frequent in older birds. Such susceptibility of embryonic tissues in older hosts to H.D. has been demonstrated and is in agreement with what has been previously noted regarding the induction of kidney carcinomas by certain viruses (Carr, 1956, 1960). Further evidence for this susceptibility of young haematopoietic tissue is afforded by observations of the blood of fowls with H.D. Such blood contains many immature leukocytes, especially of the myeloid series and a diagnosis of leukaemia would unhesitatingly be given from examination of the blood film alone. Such a response was long ago reported by Foulds (1934) with MH2 virus, and by Carr (1960), where it was given the more non-commital name "leukaemoid", for in fact these reactions may subside with increasing age if the animal survives long enough, and death is then due solely to the sarcoma. Nor can the apparently malignant blood cells induce a leukaemia following intravenous inoculation into an older animal whose haematopoietic tissue cannot show the abnormal sensitivity characteristic of the very young. In this connexion, it might be noted that Engelbreth-Holm (1933) considered the centres of extramedullary haematopoiesis as possibly participating in the leukaemia induced by the erythroleukaemia viruses, but was unable to come to any definite conclusion because of the technical difficulties of studying these areas. Furthermore, Vigier, Chouroulinkov, Oberling and Guérin (1957) showed that these areas were stimulated in the leukaemia-like reaction sometimes provoked by the GHRC/15 sarcomas, and referred to other tumours producing similar actions.

A sudden stimulation of these areas to excess multiplication of cells, many of which may be suddenly shed into the blood-stream, could easily result in a local weakening of the single layer of endothelium which usually separates them from the blood and results in an inflow of blood which does not happen in normal haematopoiesis. It may be noted that the blood pressure of birds is very much higher than that of mammals (Sturkie, 1954). This need only be temporary damage, soon repaired and not usually visible in sections of H.D. lesions, and does not imply necrosis of the endothelium. A similar explanation of invasion of the blood by tumour cells may explain the H.D. in the tumour itself.

Denial of the necrotic action of these viruses will invalidate that oft quoted linkage that they are reputed to show between the purely necrotising viruses and the proliferating ones. The desirability of such a linkage can well be questioned, and it is noteworthy that no report has ever been made on methods to elucidate the unusual virus mechanism by which these viruses had switched to induce a wholly new type of lesion. By contrast, the suggestion that the H.D. is simply another manifestation of the extended cytotropism of the viruses in young animals falls into line with all our information regarding these and the other tumour viruses, and perhaps more profitably indicates new lines of work. It also brings more closely together the sarcoma and leukaemia viruses of fowls. Many of the leukaemia viruses will also induce sarcomas (Rothe Meyer and Engelbreth-Holm, 1933; Oberling and Guérin, 1933), especially in young animals, and Benedetti (1957) showed that E.S. virus first infects the monocytes of the marrow. It is therefore not surprising that the sarcoma viruses will, in young animals, have a tendency towards leukaemia (Foulds, 1934; Carr, 1960), and as cellular differentiation becomes less and less marked with decreasing age, tend to merge. Foulds (1934) noted the Rous virus and others may tend to metastasise to the bone marrow. He was using animals of mixed ages, and did not comment on the finding noted in this work, that this is usually in very young chicks.

The study of the leukaemoid reaction induced by viruses in very young animals is not easy. Not only are the definitive haematopoietic cells involved, as in the adult, but also the earlier series of blood-forming tissues, as well as probably the extramedullary haematopoietic areas, and each is rapidly being modified as age increases. The sarcoma cells also seem to invade the blood-stream, and appear as monocytic elements. For this reason, a description of this leukaemoid state was not given in this work, as the help it might give to the present contention seemed far outweighed by the complications involved.

### SUMMARY

The tissue distribution of the Haemorrhagic Disease (H.D.) of Duran-Reynals is similar for all viruses which can induce it, and this is not compatible with the hypothesis that it is due to a necrotising action of the viruses on either normal or rapidly-proliferating endothelium. H.D. is invariably associated with areas of extramedullary haematopoiesis such as are found in many normal tissues of birds, and infection of these by the virus is regarded as the cause of the condition.

The disease can occur in embryonic tissues present in hosts too old to show the condition in their normal tissues.

All expenses in connexion with this work were borne by the British Empire Cancer Campaign.

### REFERENCES

BENEDETTI, E. L.—(1957) *Bull. Ass. franç. Cancer*, **44**, 473.
BORSOS, T. AND BANG, F. B.—(1957) *Virology*, **4**, 385.
BURMESTER, B. R., SHARPLESS, G. R. AND FONTES, A. R.—(1960) *J. nat. Cancer Inst.*, **24**, 1443.
CARR, J. G.—(1944) *Proc. Roy. Soc. Edinb.*, *B*, **62**, 51.—(1956) *Brit. J. Cancer*, **10**, 379. —(1960) *Ibid*, **14**, 77.
*Idem* AND CAMPBELL, J. G.—(1958) *Ibid*, **12**, 631.
*Idem* AND HARRIS, R. J. C.—(1951) *Ibid*, **5**, 83.
CHOUROULINKOV, I.—(1958) *Bull. Ass. franç. Cancer*, **45**, 177.
DIXON, F. J.—(1948) *Proc. Soc. exp. Biol. N.Y.*, **68**, 505.
DURAN-REYNALS, F.—(1940) *Yale J. Biol. Med.*, **13**, 77.—(1946) *Cancer Res.*, **6**, 545.
*Idem* AND ESTRADA, E.—(1940) *Proc. Soc. exp. Biol. N.Y.*, **45**, 367.
*Idem* AND SHRIGLEY, E. W.—(1946) *Cancer Res.*, **6**, 535.
ENGELBRETH-HOLM, J.—(1933) 'Experimentelle Studier over den overførbare Hønseleukose.' Copenhagen (Levin and Munksgaard).
FOULDS, L.—(1934) *Sci. Rep. Cancer Res. Fd., Lond.*, **11**, 15.
FUJINAMI, A.—(1913) *Gann*, **7**, 190.
GOLDHAFT, T. M. AND WERNIKOFF, N.—(1954) *World's Poult. Congr., Edinburgh*, **10**, 284.
MELLANBY, E.—(1938) *J. Path. Bact.*, **47**, 47.
MILFORD, J. J. AND DURAN-REYNALS, F.—(1943) *Cancer Res.*, **3**, 578.
MURRAY, J. A. AND BEGG, A. M.—(1930) *Sci. Rep. Cancer Res. Fd, Lond.*, **9**, 1.
OAKBERG, E. F.—(1950) *Poult. Sci.*, **29**, 420.
OBERLING, C. AND GUÉRIN, M.—(1933) *Bull. Ass. franç. Cancer*, **22**, 180.
PEACOCK, P. R.—(1946) *Cancer Res.*, **6**, 311.
*Idem* AND PEACOCK, A.—(1953) *Brit. J. Cancer*, **7**, 120.
ROTHE MEYER, A. AND ENGELBRETH-HOLM, J.—(1933) *Acta path. microbiol. scand.*, **10**, 380.
ROUS, P.—(1911) *J. exp. Med.*, **13**, 397.
SHARPLESS, G. R., DEFENDI, V. AND COX, H. R.—(1958) *Proc. Soc. exp. Biol. N.Y.*, **97**, 755.
SILLER, W. G. AND CARR, J. G.—(1961) *Res. vet. Sci.*, **2**, 96.
STURKIE, P. D.—(1954) 'Avian physiology'. New York. (Comstock Publishing Associates).
VIGIER, P.—(1954) *Bull. Ass. franç. Cancer*, **41**, 235.
*Idem*, CHOUROULINKOV, I., OBERLING, C. AND GUÉRIN, M.—(1957) *Bull. Ass. franç. Cancer*, **44**, 135.